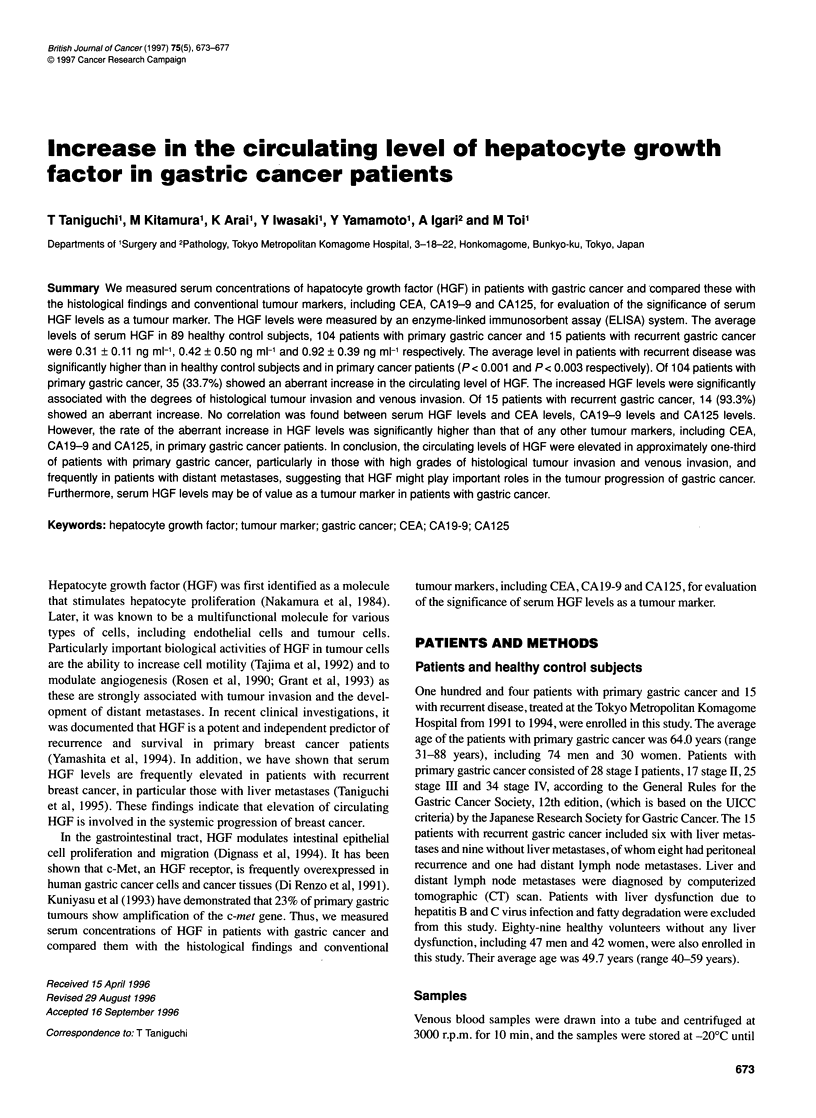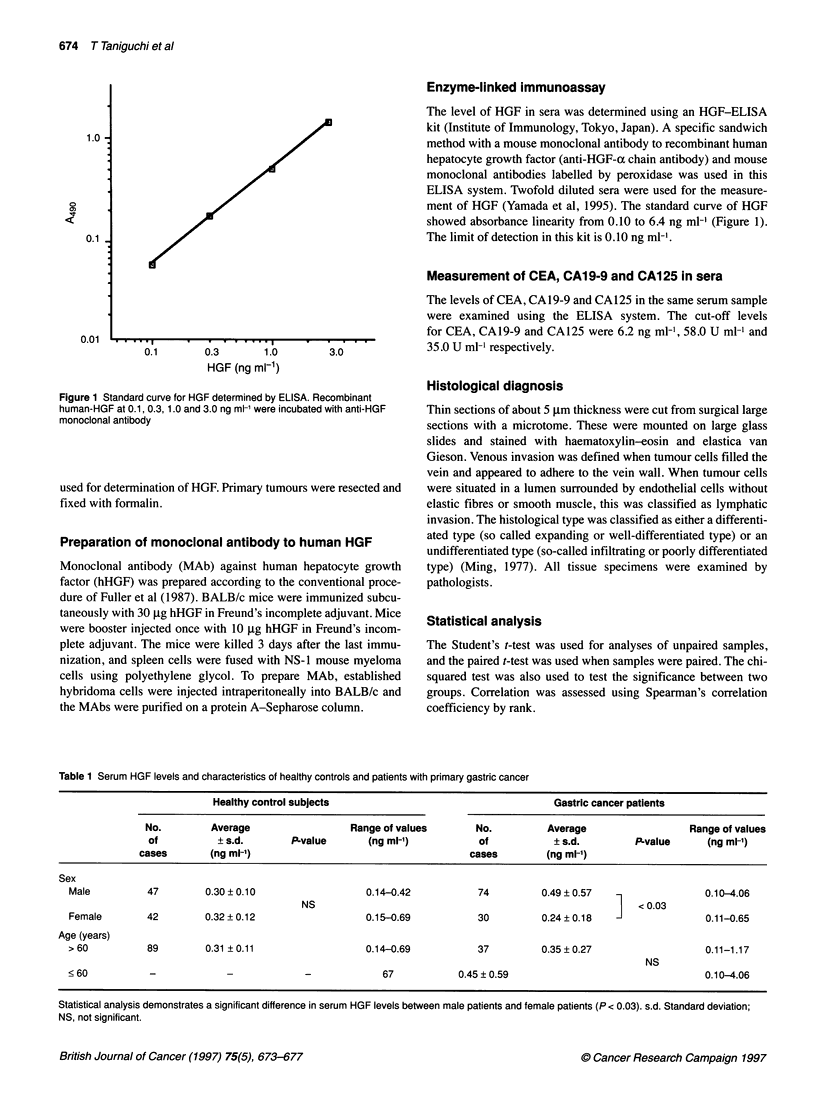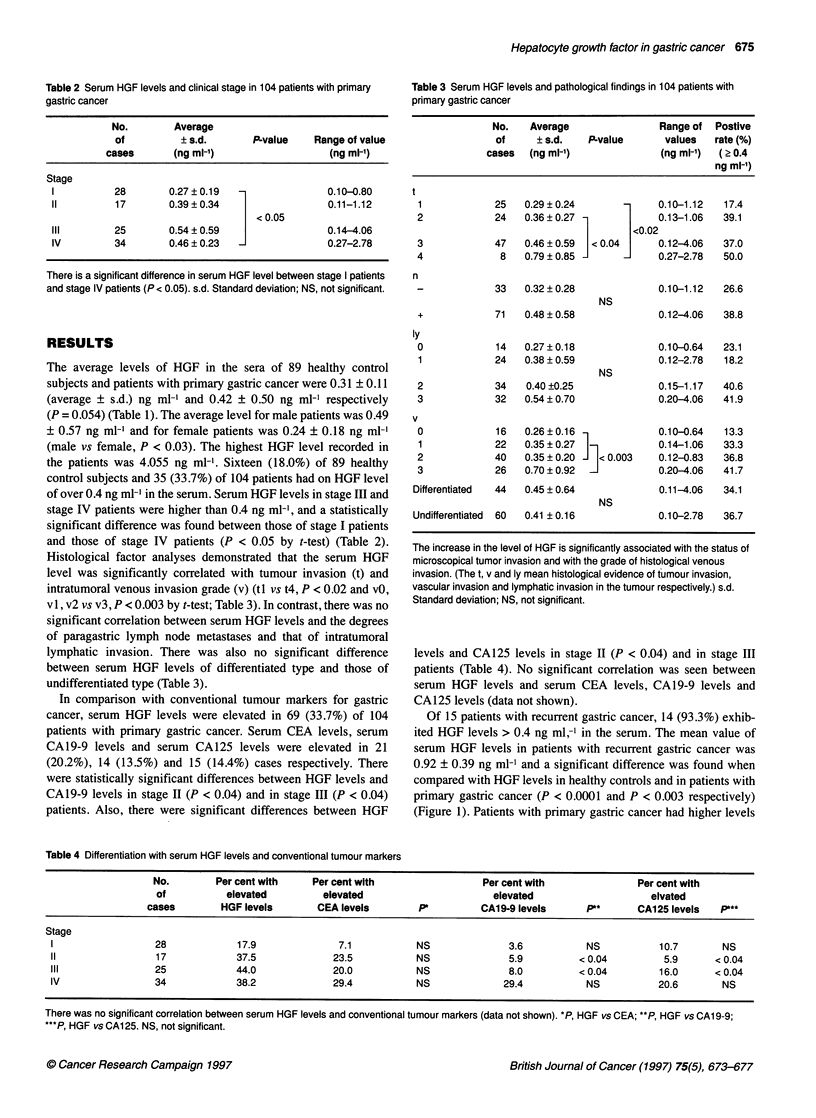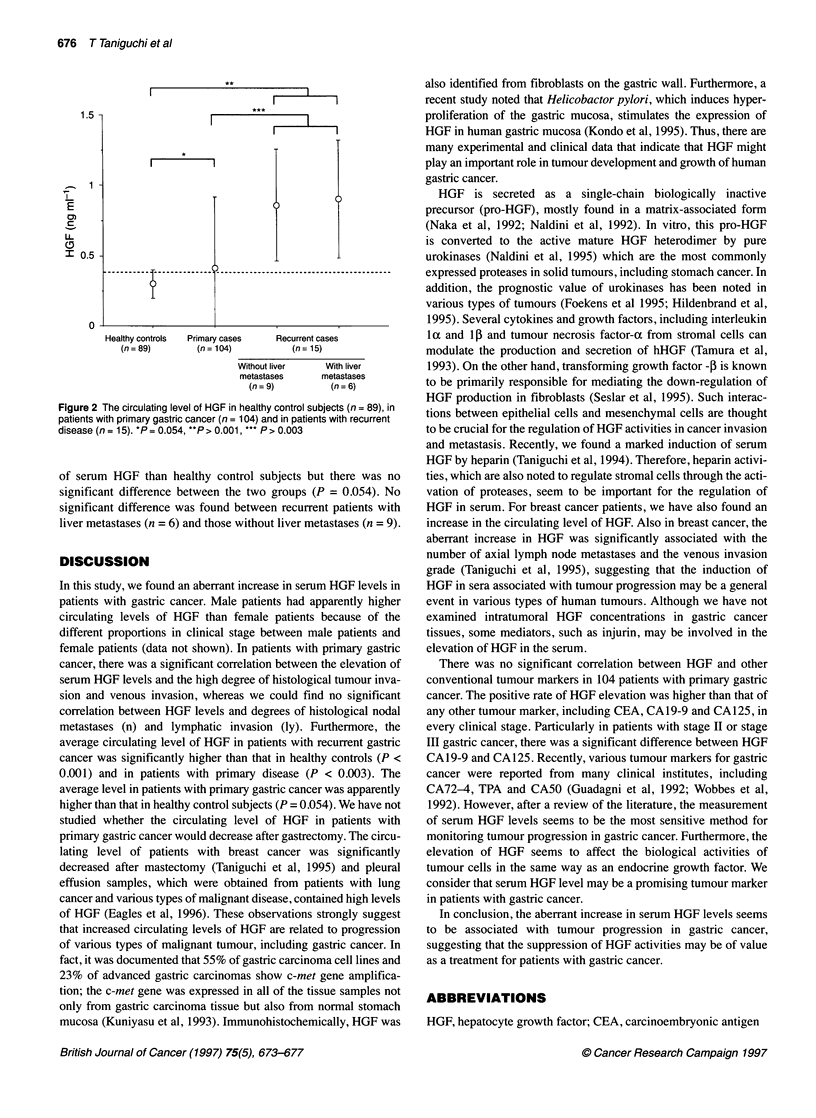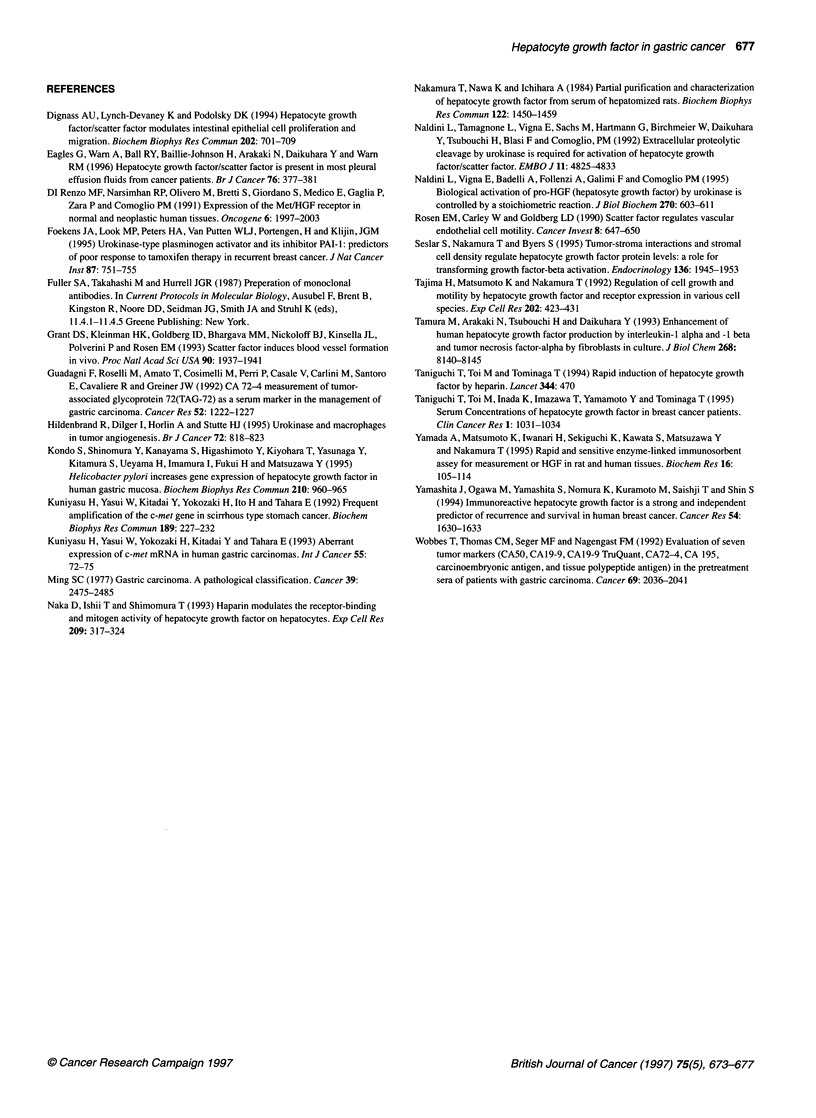# Increase in the circulating level of hepatocyte growth factor in gastric cancer patients.

**DOI:** 10.1038/bjc.1997.120

**Published:** 1997

**Authors:** T. Taniguchi, M. Kitamura, K. Arai, Y. Iwasaki, Y. Yamamoto, A. Igari, M. Toi

**Affiliations:** Department of Surgery, Tokyo Metropolitan Komagome Hospital, Bunkyo-ku, Japan.

## Abstract

We measured serum concentrations of hapatocyte growth factor (HGF) in patients with gastric cancer and compared these with the histological findings and conventional tumour markers, including CEA, CA19-9 and CA125, for evaluation of the significance of serum HGF levels as a tumour marker. The HGF levels were measured by an enzyme-linked immunosorbent assay (ELISA) system. The average levels of serum HGF in 89 healthy control subjects, 104 patients with primary gastric cancer and 15 patients with recurrent gastric cancer were 0.31 +/- 0.11 ng ml(1), 0.42 +/- 0.50 ng ml(-1) and 0.92 +/- 0.39 ng ml(-1) respectively. The average level in patients with recurrent disease was significantly higher than in healthy control subjects and in primary cancer patients (P< 0.001 and P< 0.003 respectively). Of 104 patients with primary gastric cancer, 35 (33.7%) showed an aberrant increase in the circulating level of HGF. The increased HGF levels were significantly associated with the degrees of histological tumour invasion and venous invasion. Of 15 patients with recurrent gastric cancer, 14 (93.3%) showed an aberrant increase. No correlation was found between serum HGF levels and CEA levels, CA19-9 levels and CA125 levels. However, the rate of the aberrant increase in HGF levels was significantly higher than that of any other tumour markers, including CEA, CA19-9 and CA125, in primary gastric cancer patients. In conclusion, the circulating levels of HGF were elevated in approximately one-third of patients with primary gastric cancer, particularly in those with high grades of histological tumour invasion and venous invasion, and frequently in patients with distant metastases, suggesting that HGF might play important roles in the tumour progression of gastric cancer. Furthermore, serum HGF levels may be of value as a tumour marker in patients with gastric cancer.


British Journal of Cancer (1997) 75(5), 673–677
© 1997 Cancer Research Campaign

### Increase in the circulating level of hepatocyte growth factor in gastric cancer patients

**T Taniguchi[1], M Kitamura[1], K Arai[1], Y Iwasaki[1], Y Yamamoto[1], A Igari[2] and M Toi[1]**

Departments of [1]Surgery and [2]Pathology, Tokyo Metropolitan Komagome Hospital, 3–18–22, Honkomagome, Bunkyo-ku, Tokyo, Japan

**Summary** We measured serum concentrations of hapatocyte growth factor (HGF) in patients with gastric cancer and compared these with the histological findings and conventional tumour markers, including CEA, CA19–9 and CA125, for evaluation of the significance of serum HGF levels as a tumour marker. The HGF levels were measured by an enzyme-linked immunosorbent assay (ELISA) system. The average levels of serum HGF in 89 healthy control subjects, 104 patients with primary gastric cancer and 15 patients with recurrent gastric cancer were 0.31 ± 0.11 ng ml$^{-1}$, 0.42 ± 0.50 ng ml$^{-1}$ and 0.92 ± 0.39 ng ml$^{-1}$ respectively. The average level in patients with recurrent disease was significantly higher than in healthy control subjects and in primary cancer patients ($P < 0.001$ and $P < 0.003$ respectively). Of 104 patients with primary gastric cancer, 35 (33.7%) showed an aberrant increase in the circulating level of HGF. The increased HGF levels were significantly associated with the degrees of histological tumour invasion and venous invasion. Of 15 patients with recurrent gastric cancer, 14 (93.3%) showed an aberrant increase. No correlation was found between serum HGF levels and CEA levels, CA19–9 levels and CA125 levels. However, the rate of the aberrant increase in HGF levels was significantly higher than that of any other tumour markers, including CEA, CA19–9 and CA125, in primary gastric cancer patients. In conclusion, the circulating levels of HGF were elevated in approximately one-third of patients with primary gastric cancer, particularly in those with high grades of histological tumour invasion and venous invasion, and frequently in patients with distant metastases, suggesting that HGF might play important roles in the tumour progression of gastric cancer. Furthermore, serum HGF levels may be of value as a tumour marker in patients with gastric cancer.

**Keywords:** hepatocyte growth factor; tumour marker; gastric cancer; CEA; CA19-9; CA125

Hepatocyte growth factor (HGF) was first identified as a molecule that stimulates hepatocyte proliferation (Nakamura et al, 1984). Later, it was known to be a multifunctional molecule for various types of cells, including endothelial cells and tumour cells. Particularly important biological activities of HGF in tumour cells are the ability to increase cell motility (Tajima et al, 1992) and to modulate angiogenesis (Rosen et al, 1990; Grant et al, 1993) as these are strongly associated with tumour invasion and the development of distant metastases. In recent clinical investigations, it was documented that HGF is a potent and independent predictor of recurrence and survival in primary breast cancer patients (Yamashita et al, 1994). In addition, we have shown that serum HGF levels are frequently elevated in patients with recurrent breast cancer, in particular those with liver metastases (Taniguchi et al, 1995). These findings indicate that elevation of circulating HGF is involved in the systemic progression of breast cancer.

In the gastrointestinal tract, HGF modulates intestinal epithelial cell proliferation and migration (Dignass et al, 1994). It has been shown that c-Met, an HGF receptor, is frequently overexpressed in human gastric cancer cells and cancer tissues (Di Renzo et al, 1991). Kuniyasu et al (1993) have demonstrated that 23% of primary gastric tumours show amplification of the *c-met* gene. Thus, we measured serum concentrations of HGF in patients with gastric cancer and compared them with the histological findings and conventional tumour markers, including CEA, CA19-9 and CA125, for evaluation of the significance of serum HGF levels as a tumour marker.

*Received 15 April 1996*
*Revised 29 August 1996*
*Accepted 16 September 1996*

*Correspondence to:* T Taniguchi

### PATIENTS AND METHODS

### Patients and healthy control subjects

One hundred and four patients with primary gastric cancer and 15 with recurrent disease, treated at the Tokyo Metropolitan Komagome Hospital from 1991 to 1994, were enrolled in this study. The average age of the patients with primary gastric cancer was 64.0 years (range 31–88 years), including 74 men and 30 women. Patients with primary gastric cancer consisted of 28 stage I patients, 17 stage II, 25 stage III and 34 stage IV, according to the General Rules for the Gastric Cancer Society, 12th edition, (which is based on the UICC criteria) by the Japanese Research Society for Gastric Cancer. The 15 patients with recurrent gastric cancer included six with liver metastases and nine without liver metastases, of whom eight had peritoneal recurrence and one had distant lymph node metastases. Liver and distant lymph node metastases were diagnosed by computerized tomographic (CT) scan. Patients with liver dysfunction due to hepatitis B and C virus infection and fatty degradation were excluded from this study. Eighty-nine healthy volunteers without any liver dysfunction, including 47 men and 42 women, were also enrolled in this study. Their average age was 49.7 years (range 40–59 years).

### Samples

Venous blood samples were drawn into a tube and centrifuged at 3000 r.p.m. for 10 min, and the samples were stored at –20°C until

**Figure 1** Standard curve for HGF determined by ELISA. Recombinant human-HGF at 0.1, 0.3, 1.0 and 3.0 ng ml$^{-1}$ were incubated with anti-HGF monoclonal antibody used for determination of HGF. Primary tumours were resected and fixed with formalin.

### Preparation of monoclonal antibody to human HGF

Monoclonal antibody (MAb) against human hepatocyte growth factor (hHGF) was prepared according to the conventional procedure of Fuller et al (1987). BALB/c mice were immunized subcutaneously with 30 μg hHGF in Freund's incomplete adjuvant. Mice were booster injected once with 10 μg hHGF in Freund's incomplete adjuvant. The mice were killed 3 days after the last immunization, and spleen cells were fused with NS-1 mouse myeloma cells using polyethylene glycol. To prepare MAb, established hybridoma cells were injected intraperitoneally into BALB/c and the MAbs were purified on a protein A–Sepharose column.

### Enzyme-linked immunoassay

The level of HGF in sera was determined using an HGF–ELISA kit (Institute of Immunology, Tokyo, Japan). A specific sandwich method with a mouse monoclonal antibody to recombinant human hepatocyte growth factor (anti-HGF-α chain antibody) and mouse monoclonal antibodies labelled by peroxidase was used in this ELISA system. Twofold diluted sera were used for the measurement of HGF (Yamada et al, 1995). The standard curve of HGF showed absorbance linearity from 0.10 to 6.4 ng ml$^{-1}$ (Figure 1). The limit of detection in this kit is 0.10 ng ml$^{-1}$.

### Measurement of CEA, CA19-9 and CA125 in sera

The levels of CEA, CA19-9 and CA125 in the same serum sample were examined using the ELISA system. The cut-off levels for CEA, CA19-9 and CA125 were 6.2 ng ml$^{-1}$, 58.0 U ml$^{-1}$ and 35.0 U ml$^{-1}$ respectively.

### Histological diagnosis

Thin sections of about 5 μm thickness were cut from surgical large sections with a microtome. These were mounted on large glass slides and stained with haematoxylin–eosin and elastica van Gieson. Venous invasion was defined when tumour cells filled the vein and appeared to adhere to the vein wall. When tumour cells were situated in a lumen surrounded by endothelial cells without elastic fibres or smooth muscle, this was classified as lymphatic invasion. The histological type was classified as either a differentiated type (so called expanding or well-differentiated type) or an undifferentiated type (so-called infiltrating or poorly differentiated type) (Ming, 1977). All tissue specimens were examined by pathologists.

### Statistical analysis

The Student's *t*-test was used for analyses of unpaired samples, and the paired *t*-test was used when samples were paired. The chi-squared test was also used to test the significance between two groups. Correlation was assessed using Spearman's correlation coefficiency by rank.

**Table 1** Serum HGF levels and characteristics of healthy controls and patients with primary gastric cancer

| | Healthy control subjects | | | | Gastric cancer patients | | | |
|---|---|---|---|---|---|---|---|---|
| | No. of cases | Average ± s.d. (ng ml$^{-1}$) | *P*-value | Range of values (ng ml$^{-1}$) | No. of cases | Average ± s.d. (ng ml$^{-1}$) | *P*-value | Range of values (ng ml$^{-1}$) |
| Sex | | | | | | | | |
| Male | 47 | 0.30 ± 0.10 | NS | 0.14–0.42 | 74 | 0.49 ± 0.57 | < 0.03 | 0.10–4.06 |
| Female | 42 | 0.32 ± 0.12 | | 0.15–0.69 | 30 | 0.24 ± 0.18 | | 0.11–0.65 |
| Age (years) | | | | | | | | |
| > 60 | 89 | 0.31 ± 0.11 | | 0.14–0.69 | 37 | 0.35 ± 0.27 | NS | 0.11–1.17 |
| ≤ 60 | – | – | – | 67 | 0.45 ± 0.59 | | | 0.10–4.06 |

Statistical analysis demonstrates a significant difference in serum HGF levels between male patients and female patients ($P < 0.03$). s.d. Standard deviation; NS, not significant.

© Cancer Research Campaign 1997

**Table 2** Serum HGF levels and clinical stage in 104 patients with primary gastric cancer

| | No. of cases | Average ± s.d. (ng ml$^{-1}$) | P-value | Range of value (ng ml$^{-1}$) |
|---|---|---|---|---|
| Stage | | | | |
| I | 28 | 0.27 ± 0.19 | | 0.10–0.80 |
| II | 17 | 0.39 ± 0.34 | < 0.05 | 0.11–1.12 |
| III | 25 | 0.54 ± 0.59 | | 0.14–4.06 |
| IV | 34 | 0.46 ± 0.23 | | 0.27–2.78 |

There is a significant difference in serum HGF level between stage I patients and stage IV patients ($P < 0.05$). s.d. Standard deviation; NS, not significant.

### RESULTS

The average levels of HGF in the sera of 89 healthy control subjects and patients with primary gastric cancer were 0.31 ± 0.11 (average ± s.d.) ng ml$^{-1}$ and 0.42 ± 0.50 ng ml$^{-1}$ respectively ($P = 0.054$) (Table 1). The average level for male patients was 0.49 ± 0.57 ng ml$^{-1}$ and for female patients was 0.24 ± 0.18 ng ml$^{-1}$ (male *vs* female, $P < 0.03$). The highest HGF level recorded in the patients was 4.055 ng ml$^{-1}$. Sixteen (18.0%) of 89 healthy control subjects and 35 (33.7%) of 104 patients had on HGF level of over 0.4 ng ml$^{-1}$ in the serum. Serum HGF levels in stage III and stage IV patients were higher than 0.4 ng ml$^{-1}$, and a statistically significant difference was found between those of stage I patients and those of stage IV patients ($P < 0.05$ by *t*-test) (Table 2). Histological factor analyses demonstrated that the serum HGF level was significantly correlated with tumour invasion (t) and intratumoral venous invasion grade (v) (t1 *vs* t4, $P < 0.02$ and v0, v1, v2 *vs* v3, $P < 0.003$ by *t*-test; Table 3). In contrast, there was no significant correlation between serum HGF levels and the degrees of paragastric lymph node metastases and that of intratumoral lymphatic invasion. There was also no significant difference between serum HGF levels of differentiated type and those of undifferentiated type (Table 3).

In comparison with conventional tumour markers for gastric cancer, serum HGF levels were elevated in 69 (33.7%) of 104 patients with primary gastric cancer. Serum CEA levels, serum CA19-9 levels and serum CA125 levels were elevated in 21 (20.2%), 14 (13.5%) and 15 (14.4%) cases respectively. There were statistically significant differences between HGF levels and CA19-9 levels in stage II ($P < 0.04$) and in stage III ($P < 0.04$) patients. Also, there were significant differences between HGF levels and CA125 levels in stage II ($P < 0.04$) and in stage III patients (Table 4). No significant correlation was seen between serum HGF levels and serum CEA levels, CA19-9 levels and CA125 levels (data not shown).

Of 15 patients with recurrent gastric cancer, 14 (93.3%) exhibited HGF levels > 0.4 ng ml,$^{-1}$ in the serum. The mean value of serum HGF levels in patients with recurrent gastric cancer was 0.92 ± 0.39 ng ml$^{-1}$ and a significant difference was found when compared with HGF levels in healthy controls and in patients with primary gastric cancer ($P < 0.0001$ and $P < 0.003$ respectively) (Figure 1). Patients with primary gastric cancer had higher levels

**Table 3** Serum HGF levels and pathological findings in 104 patients with primary gastric cancer

| | No. of cases | Average ± s.d. (ng ml$^{-1}$) | P-value | Range of values (ng ml$^{-1}$) | Postive rate (%) (≥ 0.4 ng ml$^{-1}$) |
|---|---|---|---|---|---|
| t | | | | | |
| 1 | 25 | 0.29 ± 0.24 | | 0.10–1.12 | 17.4 |
| 2 | 24 | 0.36 ± 0.27 | <0.02 | 0.13–1.06 | 39.1 |
| 3 | 47 | 0.46 ± 0.59 | < 0.04 | 0.12–4.06 | 37.0 |
| 4 | 8 | 0.79 ± 0.85 | | 0.27–2.78 | 50.0 |
| n | | | | | |
| – | 33 | 0.32 ± 0.28 | NS | 0.10–1.12 | 26.6 |
| + | 71 | 0.48 ± 0.58 | | 0.12–4.06 | 38.8 |
| ly | | | | | |
| 0 | 14 | 0.27 ± 0.18 | | 0.10–0.64 | 23.1 |
| 1 | 24 | 0.38 ± 0.59 | NS | 0.12–2.78 | 18.2 |
| 2 | 34 | 0.40 ±0.25 | | 0.15–1.17 | 40.6 |
| 3 | 32 | 0.54 ± 0.70 | | 0.20–4.06 | 41.9 |
| v | | | | | |
| 0 | 16 | 0.26 ± 0.16 | | 0.10–0.64 | 13.3 |
| 1 | 22 | 0.35 ± 0.27 | | 0.14–1.06 | 33.3 |
| 2 | 40 | 0.35 ± 0.20 | < 0.003 | 0.12–0.83 | 36.8 |
| 3 | 26 | 0.70 ± 0.92 | | 0.20–4.06 | 41.7 |
| Differentiated | 44 | 0.45 ± 0.64 | NS | 0.11–4.06 | 34.1 |
| Undifferentiated | 60 | 0.41 ± 0.16 | | 0.10–2.78 | 36.7 |

The increase in the level of HGF is significantly associated with the status of microscopical tumor invasion and with the grade of histological venous invasion. (The t, v and ly mean histological evidence of tumour invasion, vascular invasion and lymphatic invasion in the tumour respectively.) s.d. Standard deviation; NS, not significant.

**Table 4** Differentiation with serum HGF levels and conventional tumour markers

| | No. of cases | Per cent with elevated HGF levels | Per cent with elevated CEA levels | P* | Per cent with elevated CA19-9 levels | P** | Per cent with elvated CA125 levels | P*** |
|---|---|---|---|---|---|---|---|---|
| Stage | | | | | | | | |
| I | 28 | 17.9 | 7.1 | NS | 3.6 | NS | 10.7 | NS |
| II | 17 | 37.5 | 23.5 | NS | 5.9 | < 0.04 | 5.9 | < 0.04 |
| III | 25 | 44.0 | 20.0 | NS | 8.0 | < 0.04 | 16.0 | < 0.04 |
| IV | 34 | 38.2 | 29.4 | NS | 29.4 | NS | 20.6 | NS |

There was no significant correlation between serum HGF levels and conventional tumour markers (data not shown). *P, HGF *vs* CEA; **P, HGF *vs* CA19-9; ***P, HGF *vs* CA125. NS, not significant.

© Cancer Research Campaign 1997

**Figure 2** The circulating level of HGF in healthy control subjects ($n$ = 89), in patients with primary gastric cancer ($n$ = 104) and in patients with recurrent disease ($n$ = 15). $^{*}P$ = 0.054, $^{**}P$ > 0.001, $^{***}$ $P$ > 0.003

of serum HGF than healthy control subjects but there was no significant difference between the two groups ($P$ = 0.054). No significant difference was found between recurrent patients with liver metastases ($n$ = 6) and those without liver metastases ($n$ = 9).

### DISCUSSION

In this study, we found an aberrant increase in serum HGF levels in patients with gastric cancer. Male patients had apparently higher circulating levels of HGF than female patients because of the different proportions in clinical stage between male patients and female patients (data not shown). In patients with primary gastric cancer, there was a significant correlation between the elevation of serum HGF levels and the high degree of histological tumour invasion and venous invasion, whereas we could find no significant correlation between HGF levels and degrees of histological nodal metastases (n) and lymphatic invasion (ly). Furthermore, the average circulating level of HGF in patients with recurrent gastric cancer was significantly higher than that in healthy controls ($P$ < 0.001) and in patients with primary disease ($P$ < 0.003). The average level in patients with primary gastric cancer was apparently higher than that in healthy control subjects ($P$ = 0.054). We have not studied whether the circulating level of HGF in patients with primary gastric cancer would decrease after gastrectomy. The circulating level of patients with breast cancer was significantly decreased after mastectomy (Taniguchi et al, 1995) and pleural effusion samples, which were obtained from patients with lung cancer and various types of malignant disease, contained high levels of HGF (Eagles et al, 1996). These observations strongly suggest that increased circulating levels of HGF are related to progression of various types of malignant tumour, including gastric cancer. In fact, it was documented that 55% of gastric carcinoma cell lines and 23% of advanced gastric carcinomas show c-*met* gene amplification; the c-*met* gene was expressed in all of the tissue samples not only from gastric carcinoma tissue but also from normal stomach mucosa (Kuniyasu et al, 1993). Immunohistochemically, HGF was also identified from fibroblasts on the gastric wall. Furthermore, a recent study noted that *Helicobactor pylori*, which induces hyperproliferation of the gastric mucosa, stimulates the expression of HGF in human gastric mucosa (Kondo et al, 1995). Thus, there are many experimental and clinical data that indicate that HGF might play an important role in tumour development and growth of human gastric cancer.

HGF is secreted as a single-chain biologically inactive precursor (pro-HGF), mostly found in a matrix-associated form (Naka et al, 1992; Naldini et al, 1992). In vitro, this pro-HGF is converted to the active mature HGF heterodimer by pure urokinases (Naldini et al, 1995) which are the most commonly expressed proteases in solid tumours, including stomach cancer. In addition, the prognostic value of urokinases has been noted in various types of tumours (Foekens et al 1995; Hildenbrand et al, 1995). Several cytokines and growth factors, including interleukin 1$\alpha$ and 1$\beta$ and tumour necrosis factor-$\alpha$ from stromal cells can modulate the production and secretion of hHGF (Tamura et al, 1993). On the other hand, transforming growth factor -$\beta$ is known to be primarily responsible for mediating the down-regulation of HGF production in fibroblasts (Seslar et al, 1995). Such interactions between epithelial cells and mesenchymal cells are thought to be crucial for the regulation of HGF activities in cancer invasion and metastasis. Recently, we found a marked induction of serum HGF by heparin (Taniguchi et al, 1994). Therefore, heparin activities, which are also noted to regulate stromal cells through the activation of proteases, seem to be important for the regulation of HGF in serum. For breast cancer patients, we have also found an increase in the circulating level of HGF. Also in breast cancer, the aberrant increase in HGF was significantly associated with the number of axial lymph node metastases and the venous invasion grade (Taniguchi et al, 1995), suggesting that the induction of HGF in sera associated with tumour progression may be a general event in various types of human tumours. Although we have not examined intratumoral HGF concentrations in gastric cancer tissues, some mediators, such as injurin, may be involved in the elevation of HGF in the serum.

There was no significant correlation between HGF and other conventional tumour markers in 104 patients with primary gastric cancer. The positive rate of HGF elevation was higher than that of any other tumour marker, including CEA, CA19-9 and CA125, in every clinical stage. Particularly in patients with stage II or stage III gastric cancer, there was a significant difference between HGF CA19-9 and CA125. Recently, various tumour markers for gastric cancer were reported from many clinical institutes, including CA72–4, TPA and CA50 (Guadagni et al, 1992; Wobbes et al, 1992). However, after a review of the literature, the measurement of serum HGF levels seems to be the most sensitive method for monitoring tumour progression in gastric cancer. Furthermore, the elevation of HGF seems to affect the biological activities of tumour cells in the same way as an endocrine growth factor. We consider that serum HGF level may be a promising tumour marker in patients with gastric cancer.

In conclusion, the aberrant increase in serum HGF levels seems to be associated with tumour progression in gastric cancer, suggesting that the suppression of HGF activities may be of value as a treatment for patients with gastric cancer.

### ABBREVIATIONS

HGF, hepatocyte growth factor; CEA, carcinoembryonic antigen

© Cancer Research Campaign 1997

### REFERENCES

Dignass AU, Lynch-Devaney K and Podolsky DK (1994) Hepatocyte growth factor/scatter factor modulates intestinal epithelial cell proliferation and migration. *Biochem Biophys Res Commun* **202**: 701–709

Eagles G, Warn A, Ball RY, Baillie-Johnson H, Arakaki N, Daikuhara Y and Warn RM (1996) Hepatocyte growth factor/scatter factor is present in most pleural effusion fluids from cancer patients. *Br J Cancer* **76**: 377–381

DI Renzo MF, Narsimhan RP, Olivero M, Bretti S, Giordano S, Medico E, Gaglia P, Zara P and Comoglio PM (1991) Expression of the Met/HGF receptor in normal and neoplastic human tissues. *Oncogene* **6**: 1997–2003

Foekens JA, Look MP, Peters HA, Van Putten WLJ, Portengen, H and Klijin, JGM (1995) Urokinase-type plasminogen activator and its inhibitor PAI-1: predictors of poor response to tamoxifen therapy in recurrent breast cancer. *J Nat Cancer Inst* **87**: 751–755

Fuller SA, Takahashi M and Hurrell JGR (1987) Preperation of monoclonal antibodies. In *Current Protocols in Molecular Biology*, Ausubel F, Brent B, Kingston R, Noore DD, Seidman JG, Smith JA and Struhl K (eds), 11.4.1–11.4.5 Greene Publishing: New York.

Grant DS, Kleinman HK, Goldberg ID, Bhargava MM, Nickoloff BJ, Kinsella JL, Polverini P and Rosen EM (1993) Scatter factor induces blood vessel formation in vivo. *Proc Natl Acad Sci USA* **90**: 1937–1941

Guadagni F, Roselli M, Amato T, Cosimelli M, Perri P, Casale V, Carlini M, Santoro E, Cavaliere R and Greiner JW (1992) CA 72–4 measurement of tumor-associated glycoprotein 72(TAG-72) as a serum marker in the management of gastric carcinoma. *Cancer Res* **52**: 1222–1227

Hildenbrand R, Dilger I, Horlin A and Stutte HJ (1995) Urokinase and macrophages in tumor angiogenesis. *Br J Cancer* **72**: 818–823

Kondo S, Shinomura Y, Kanayama S, Higashimoto Y, Kiyohara T, Yasunaga Y, Kitamura S, Ueyama H, Imamura I, Fukui H and Matsuzawa Y (1995) *Helicobacter pylori* increases gene expression of hepatocyte growth factor in human gastric mucosa. *Biochem Biophys Res Commun* **210**: 960–965

Kuniyasu H, Yasui W, Kitadai Y, Yokozaki H, Ito H and Tahara E (1992) Frequent amplification of the c-*met* gene in scirrhous type stomach cancer. *Biochem Biophys Res Commun* **189**: 227–232

Kuniyasu H, Yasui W, Yokozaki H, Kitadai Y and Tahara E (1993) Aberrant expression of c-*met* mRNA in human gastric carcinomas. *Int J Cancer* **55**: 72–75

Ming SC (1977) Gastric carcinoma. A pathological classification. *Cancer* **39**: 2475–2485

Naka D, Ishii T and Shimomura T (1993) Haparin modulates the receptor-binding and mitogen activity of hepatocyte growth factor on hepatocytes. *Exp Cell Res* **209:** 317–324

Nakamura T, Nawa K and Ichihara A (1984) Partial purification and characterization of hepatocyte growth factor from serum of hepatomized rats. *Biochem Biophys Res Commun* **122**: 1450–1459

Naldini L, Tamagnone L, Vigna E, Sachs M, Hartmann G, Birchmeier W, Daikuhara Y, Tsubouchi H, Blasi F and Comoglio, PM (1992) Extracellular proteolytic cleavage by urokinase is required for activation of hepatocyte growth factor/scatter factor. *EMBO J* **11**: 4825–4833

Naldini L, Vigna E, Badelli A, Follenzi A, Galimi F and Comoglio PM (1995) Biological activation of pro-HGF (hepatosyte growth factor) by urokinase is controlled by a stoichiometric reaction. *J Biol Biochem* **270**: 603–611

Rosen EM, Carley W and Goldberg LD (1990) Scatter factor regulates vascular endothelial cell motility. *Cancer Invest* **8**: 647–650

Seslar S, Nakamura T and Byers S (1995) Tumor-stroma interactions and stromal cell density regulate hepatocyte growth factor protein levels: a role for transforming growth factor-beta activation. *Endocrinology* **136**: 1945–1953

Tajima H, Matsumoto K and Nakamura T (1992) Regulation of cell growth and motility by hepatocyte growth factor and receptor expression in various cell species. *Exp Cell Res* **202**: 423–431

Tamura M, Arakaki N, Tsubouchi H and Daikuhara Y (1993) Enhancement of human hepatocyte growth factor production by interleukin-1 alpha and -1 beta and tumor necrosis factor-alpha by fibroblasts in culture. *J Biol Chem* **268:** 8140–8145

Taniguchi T, Toi M and Tominaga T (1994) Rapid induction of hepatocyte growth factor by heparin. *Lancet* **344**: 470

Taniguchi T, Toi M, Inada K, Imazawa T, Yamamoto Y and Tominaga T (1995) Serum Concentrations of hepatocyte growth factor in breast cancer patients. *Clin Cancer Res* **1**: 1031–1034

Yamada A, Matsumoto K, Iwanari H, Sekiguchi K, Kawata S, Matsuzawa Y and Nakamura T (1995) Rapid and sensitive enzyme-linked immunosorbent assey for measurement or HGF in rat and human tissues. *Biochem Res* **16**: 105–114

Yamashita J, Ogawa M, Yamashita S, Nomura K, Kuramoto M, Saishji T and Shin S (1994) Immunoreactive hepatocyte growth factor is a strong and independent predictor of recurrence and survival in human breast cancer. *Cancer Res* **54**: 1630–1633

Wobbes T, Thomas CM, Seger MF and Nagengast FM (1992) Evaluation of seven tumor markers (CA50, CA19-9, CA19-9 TruQuant, CA72–4, CA 195, carcinoembryonic antigen, and tissue polypeptide antigen) in the pretreatment sera of patients with gastric carcinoma. *Cancer* **69**: 2036–2041

© Cancer Research Campaign 1997